# Immigrant Healthcare Experiences and Impacts During COVID-19: A Cross-Sectional Study in Alberta, Canada

**DOI:** 10.1177/23743735221112707

**Published:** 2022-07-11

**Authors:** Bishnu Bahadur Bajgain, Jeanette Jackson, Fariba Aghajafari, Carmelle Bolo, Maria-Jose Santana

**Affiliations:** 1 Department of Community Health Sciences, 70401 Cumming School of Medicine, University of Calgary, Calgary, Alberta, Canada; 2 152250 Health Quality Council of Alberta, Calgary, Alberta, Canada; 3 Department of Family Medicine and Community Health Sciences, 70401 Cumming School of Medicine, University of Calgary, Calgary, Alberta, Canada

**Keywords:** cross-sectional study, COVID-19, pandemic, primary health care, mental health, virtual care, vaccine, immigrants

## Abstract

Primary Health Care is a gateway of healthcare services. The COVID-19 pandemic has modified the process of delivering care. We aimed to assess Albertan's healthcare experiences during the pandemic and compared experiences between Albertans that were born in and outside Canada. A cross-sectional online survey (experiences and impacts of COVID-19) was conducted in October 2020, 16 years, and older Albertans. Descriptive statistics and multivariable logistic regression were performed using STATA. Of 10 175 study participants, 10% were born outside Canada. Demographics were significantly different between the 2 groups (*P* < .05). Results revealed that Canadian-born were more likely to report worse mental and physical health status (AOR = 1.36; 95% CI: 1.17-1.56), and higher stress, anxiety, and depression during the pandemic (AOR = 1.37; 95% CI: 1.16-1.60) than those born outside Canada. However, Canadian-born reported a trend toward better virtual care experiences (AOR = 1.16; 95% CI: 0.94-1.44). Albertans reported negative health impacts due to delay in care during the pandemic and vaccine hesitancy for COVID-19, which was not significantly difference among the cohorts (*P* > .05). The study findings can inform primary healthcare providers and policymakers that could be used to develop quality improvement strategies.

## Introduction

For most healthcare systems, Primary Health Care (PHC) offers patients the first contact to access medical care (1). In Canada, PHC is the gateway of healthcare services, which coordinates patients’ health care services to ensure continuity of care and ease of movement across the healthcare system when more specialized services are needed (1). PHC is linked with improved access to care, reduction in health inequalities, and better outcomes at lower cost (2). PHC is the foundation of effective health systems providing continuous quality healthcare to patients.

The COVID-19 pandemic modified the process of delivering PHC (3). As of August 5, 2021, Canada reported over 1.4 million (1 436 641) cases including 26 637 life losses due to COVID-19. Alberta has the third-most cases of COVID-19 in Canada, 235 641 confirmed cases and 2329 deaths (4, 5). Thus, in order to ensure safety and quality service delivery during the pandemic, PHC needed to strategize and reorganize services including allocation of resources (eg, manpower, infrastructure, technology and supplies, financial support); providing an up to date knowledge about technology/virtual care process to healthcare providers to meet the demand of care; implementing appropriate protocols and appropriate communication channels while strengthening care delivery processes in a timely manner (6, 7). One of the adaptations was the uptake of virtual care, switching in-person care into virtual visit such as, phone call, videoconference, and secure messages (3–9).

Due to these adaptations in care, it is crucial to understand patients’ experiences in accessing and receiving care during the pandemic. The objective of this paper is to understand patient experiences during the pandemic and compare experiences among individuals born in Canada and outside of Canada (eg, first-generation immigrants, refugees, and newcomers) (10). This work has been done in partnership with the Health Quality Council of Alberta (HQCA).

## Methods

### Study Design and Setting

This is a cross-sectional study using a provincial survey, “COVID-19 Experiences, and Impact Survey.” Albertans were surveyed to share their experiences with the health care system and the impact of the pandemic on accessing and receiving PHC. The survey also included questions on self-reported physical and mental health status, accessing and receiving virtual care experiences, and people's views on vaccination.

### Survey Tool

The survey tool was developed based on a national and international environmental scan conducted by HQCA in April 2020. The survey questionnaire was discussed, tested, and refined by 12 members of the HQCA's Patient and Family Advisory Committee. These advisors work with HQCA on issues related to patient safety, person-centered care, and quality issues from patients, and family's perspectives (APPENDIX 1 FOR SURVEY).

### Study Population and Data Collection

The study population included adults living in Alberta, Canadian-born and born outside Canada (10), who have experienced care for COVID-19, or other illnesses during the pandemic. Individuals born outside Canada include first-generation immigrants, refugees, and newcomers (10). As the survey was in English and administered online, those who were able to read and write in English and had the means to access the survey participated in this study. The study sample was quite representative of both cohorts, as around one-fifth of Albertans (21.2%, [29.4% Calgarians]) were born outside Canada (11), and so with 21.9% of the Canadian population (12).

Recruitment was undertaken via email invitation to over 15 000 Albertans, social media (Facebook, Twitter, and LinkedIn), the HQCA website, as well as advertising in the daily COVID-19 news updates by the Chief Medical Officer of Health in Alberta. The data collection occurred from April to October 2020, at the height of the pandemic (second wave) in Alberta. The participants’ informed consent was obtained via online at the start of the survey. The consent form clarified the objectives of the survey, the expected time taken to complete it, the privacy and confidentiality of the information, and the voluntary participation in the survey. The survey also included participants’ socio-demographic information (including country of birth), self-reported physical and mental health status, and questions related to vaccine hesitancy.

### Statistical Analysis

Descriptive statistical analysis was performed on the categorical data and presented as frequencies and percentages. A sample weight was calculated and applied to represent Albertans on age, gender, and 5 Alberta Health Services Zones. The chi-squared test was applied to observe the sociodemographic characteristics and the relationship between the 2 groups of interest: (1) Canadian-born individuals and (2) Individuals born outside Canada.

Multivariable logistic regression was employed to establish the adjusted odds ratios (AOR) and 95% confidence intervals (95% CI) of the predictors of the relationship between “Canadian-born” or “born outside Canadian” and outcomes. Analysis was adjusted for age, gender, education, language, income, and financial situation. All outcome variables were dichotomized. Self-reported physical and mental health status was measured on a 5-item Likert scale from much worse to much better and dichotomized to worse (slightly worse, much worse) and better or same (about the same, slightly better, and much better) health. All the analyses were performed using STATA Version 14.2, and *P*-values <.05 were considered statistically significant. The cohort of individuals born outside Canada was used as a reference group for the logistic regression analysis. The outcomes of interest include self-reported health status; impact on health due to care delay, virtual care experiences, virtual care as a good alternative for future healthcare, and vaccination hesitancy for COVID-19.

## Results

### Survey Respondents’ Demographics Characteristics

In total, 10 175 surveys were collected during October 2020. Nearly 10% (1042) survey respondents reported their status as born outside Canada. Table 1 represents the sociodemographic characteristics of the survey participants. Two-thirds (66.75%) of the participants were between 35 and 64 years old. Similarly, most participants (72.14%) were female and had at least a “college” or “university” degree (86.82%). Of 10 008 who shared information about their financial situation, 6896 (68.90%) reported a comfortable financial situation. Over three-quarters of participants reported their yearly household income as $150 000 or lower (n = 7033; 76.77%). Most of the survey respondents (98.28%) spoke English at home (Table 1). All survey respondent characteristics, with the exception to gender, showed significant difference between Canadian-born and born outside Canada (*P* < .05).

**Table 1.** Sociodemographic Characteristics of the Study Population According to Canadian-Born and Born Outside Canada.

Characteristics		Canadian-born	Born outside Canada	*P*-value
	Total N (%)	n (%)	n (%)	
**Age**	10 175	9133 (89.76)	1042 (10.24)	.0001
16-34 years	1473 (14.48)	1341 (14.68)	132 (12.67)	
35-64 years	6792 (66.75)	6124 (67.05)	668 (64.11)	
65 + years	1910 (18.77)	1668 (18.26)	242 (23.22)	
**Gender**	10 077	9043	1034	.057
Female	7270 (72.14)	6550 (72.40)	720 (69.60)	
Male	2807 (27.86)	2493 (27.60)	314 (30.4)	
**Educational attainment**	10 159	9120	1039	.0001
High school	1339 (13.18)	1224 (13.40)	115 (11.10)	
College	4483 (44.13)	4103 (45.00)	380 (36.60)	
University	4337 (42.69)	3793 (41.60)	544 (52.40)	
**Language spoken at home**	10 234	9185	1049	.0001
English	10 058 (98.28)	9140 (99.50)	918 (87.50)	
Other	176 (1.72)	45 (0.50)	131 (12.50)	
**Yearly household income**	9161	6225	936	.031
$150,000 and below	7033 (76.77)	6288 (68.88)	745 (79.60)	
Above $150,000	2128 (23.23)	1937 (31.12)	191 (20.40)	
**Financial situation**	10 008	8982	1026	.0001
Comfortable	6896 (68.90)	6129 (68.24)	767 (74.76)	
Tight	3112 (31.10)	2853 (31.76)	259 (25.24)	

### Factors Influencing Patient's Experiences in Receiving Care

Table 2 shows both, unadjusted and adjusted odds ratios (OR vs. AOR) for the factors that influence patient experiences in receiving care using multivariable logistic regression analysis. After adjustment for age, gender, education, language, income, and financial situation. Overall, Canadian-born individuals were more likely to report their mental and physical health status as “worse” or “much worse” during COVID-19 compared to individuals born outside of Canada (65% vs 56%; AOR = 1.36; 95% CI: 1.17-1.56). Similarly, the survey findings also showed a higher likelihood of perceived stress, anxiety, or depression among individuals born in Canada (75% vs 66%; AOR = 1.37; 95% CI: 1.16-1.60). Albertans (both born in Canada and outside) reported their health was negatively impacted due to delay in care (27% vs 27%; AOR = 0.89; 95% CI: 0.75-1.06) since the onset of the pandemic, which was not significantly different among the cohorts (*P* > .05). Equally, vaccine hesitancy for COVID-19 was presented in both cohorts (29% vs 29%; AOR = 0.91; 95% CI: 0.69-1.21), and it was not statistically different between individuals born in Canadian and those born outside Canada (*P* > .05). Similarly, there was a trend toward better overall virtual care experiences among Canadian-born individuals (61% vs 58%; AOR = 1.16; 95% CI: 0.94-1.44). Interestingly, Canadian-born slightly favored virtual healthcare visits over in-person visits as a resulting adoption of the pandemic (58% vs 52%; AOR = 1.22; 95% CI: 1.06-1.42).

**Table 2.** Unadjusted and Adjusted Odds Ratios (OR, AOR, 95% confidence interval [CI]) of Patient Experiences, Compared Between 2 Cohorts: Canadian-Born and Born Outside Canada.

Outcomes	OR (95% CI)	AOR (95% CI)^a^
Mental health worsened (worse or much worse)	1.45 (1.27-1.65)^b^	1.36 (1.17-1.56)^b^
Increased stress, anxiety, depression, and difficulty to cope with it	2.13 (1.87-2.43)^b^	1.37 (1.16-1.60)^b^
Physical health worsened (worse or much worse)	1.45 (1.27-1.65)^b^	1.36 (1.17-1.58)^b^
Impacted due to delay in healthcare	1.02 (0.79-1.30)	0.91 (0.68-1.21)
Rating of Virtual care experiences (top of scale 8-10)	1.13 (0.93-1.36)	1.16 (0.94-1.44)
Virtual healthcare visits as a good alternative to in-person for future healthcare	1.25 (1.10-1.42)^b^	1.22 (1.06-1.42)^b^
Would not choose to get vaccinated for COVID-19	1.01 (0.87-1.16)	0.89 (0.75-1.06)

^a^ OR adjusted for age, gender, education, language, income, and financial situation, as well as displayed for those who were born in Canada (reference category) compared to those who were born outside Canada.

^b^ Significantly different from individuals born outside Canada (*P* < .05).

## Discussion

This study reports the experiences of Albertans during COVID-19. When comparing the health status and experiences of Canadian-born with those born outside Canada, there were differences specifically related to mental and physical health and perceptions on receiving virtual care, vaccine hesitancy, and health impact due to delay in care during the pandemic. Mental health has been adversely impacted since the onset of COVID-19; increased stress, anxiety, and depression were observed, specifically higher among Canadian-born. Likewise, Canadian-born reported a better virtual care experience and believed that it could be an alternative to in-person care compared to individuals born outside Canada. Moreover, since the onset of the COVID-19, Albertan's health was negatively impacted due to delay in care, and no difference was presented between individuals born in Canada and those born outside Canada. Likely, vaccine hesitancy for COVID-19 was no different in both cohorts.

Globally, as COVID-19 affected healthcare access/delivery, Canada adopted a highly intensive intervention with the aim of reducing the severity of the pandemic (lockdown, social distancing, substantially ramping down all elective surgeries, and nonessential health services), and promoted virtual care visits (13). An ongoing COVID-19 related survey among primary care providers in Canada and United States revealed a widespread uptake of virtual care: phone calls, video conferences, and secure text message (14, 15). Canada widely switched in-person care into virtual visit for individuals who frequently needed care and in-person care was not essential: for people living with chronic physical and mental health conditions (3–9). Despite some disadvantages of virtual care visits, for example, the inability to perform medical procedures/physical examination, missing visual clues, and difficulty establishing therapeutic relationships, a study found that Canadians seem to be highly satisfied with virtual, and over one-third (38%) would choose virtual care to be the first point of contact post-COVID-19 (16), our results partly corroborated this finding specifically for people born in Canada. However, people born outside Canada did not favor virtual care and coped with it as a temporary solution, and this could be due to proficiency in speaking English.

Our findings aligned with the previous study (17, 18) that found Canadian's mental health has been negatively impacted since the onset of the COVID-19, and individuals struggled with uncertainty, fear about their own/loved one's health, employment/financial concerns, and public health guidelines (17, 18) however, in our study this is different for individuals born outside of Canada. Our findings show that individuals born in Canada reported higher mental health concerns during the COVID-19 compared to those born outside Canada, further research would be needed to understand if individuals born outside Canada were unwilling to share their mental health issues such as socio-cultural stigma. Our findings also corroborate the previous poll (19) that 50% of Canadian stated worsened mental health since the pandemic started, feeling worried (44%), and anxious (41%), including women, young, and families with small children, who lost jobs, worried about the financial situation (20).

Delay in seeking care might be due to the combination of availability of services and fearing exposure to COVID-19, which might lead towards increasing the risk of morbidity and mortality associated with a preventable/treatable health condition. Our results (33%) coincide with previous research that found almost 4 in 10 Canadians with chronic health conditions (38%), reported avoiding accessing healthcare (21). Chen-See showed that 54% of patients with cancer either canceled, postponed, or rescheduled their appointment during the pandemic (22), which was also reported (41%) in a study among adults in the United States (23), and 74% of those with delayed care had a major impact on their mental and emotional wellbeing (22).

Mass vaccination for COVID-19 is crucial. A result from the Canadian Community Health Survey revealed that 76.9% of Canadians were either very or somewhat willing to get vaccinated against COVID-19 (24). Our result also supports the sequence of earlier findings regarding vaccine hesitancy for COVID-19 (29%), as the previous study also found that 14% of Canadians indicated they would not get vaccinated (25). A recent study conducted based on twitter respondents reported that vaccine hesitancy stemmed from safety concerns, suspicion of political or economic force during the pandemic, lack of knowledge about vaccines, antivaccine or confusing messages from an authority, and lack of legal liability from vaccine companies (26).

Equity in healthcare in an inclusive environment that responds to diversity in a population is a challenge that needs a big commitment and is only achieved when everyone has the opportunity to access and receive healthcare with their own identity, culture, and characteristics without discrimination or barriers (27, 28). Evidence shows that underrepresented populations including Indigenous, immigrants, refugees, visible minorities, French-speaking Albertans, and people experiencing homelessness reported poor health and barriers to accessing healthcare services (28). Healthcare organizations are facing challenges to approach vulnerable individuals and address those issues (28). Thus, identifying individuals at high risk, the barriers they might be facing, and providing evidence-based support and treatment would be beneficial for promoting/preventing declines in an individual's health status. A recent review showed that immigrants encountered various challenges including language, culture, socioeconomic, and healthcare structure in accessing PHC (29). However, an earlier study revealed that immigrants reported equal access to primary care compared to Canadian-born, but their primary care visits were 5.3 times more frequent than Canadian-born (30). Our study reveals Canadian-born experienced higher rates of mental and physical health issues since the onset of the pandemic compared to individuals born outside Canada, whereas the impact on health due to delay in care was reported among both cohorts. People might be nervous and avoid seeking care during the pandemic because they do not want to be exposed to COVID-19 in medical facilities. Establishing strong communication strategies about the safety protocol between patients and care providers and offering virtual care options might be the key to encouraging patients to seek out care and combat the negative impacts of delayed care.

### Strengths/Limitations

The strength of this cross-sectional study is the representative sample of Albertans providing their healthcare experiences and the timing of data collection.

This study also has some constraints. We acknowledge that the study population ratio between the 2 cohorts was different, but the sample was quite representative, as around one-fifth of Albertans (21.2%, [29.4% Calgarians]) were born outside Canada (11), and as with 21.9% of the Canadian population (12). As well, we also admit that the survey may have captured a select group of individuals (the survey was offered in English solely and in an online version) likely introducing selection bias and limiting the generalizability, but a wider representation of the population (such as non-English speaking individuals, or/and English speaking but did not have the means to access the survey, not quite settled to understand how to access the survey, people working more than one job (or longer shift) and did not have time to participate in the survey, and individuals in working-class including meat plants) would have provided added evidence. We also concede that self-reported measures are subject to recall bias and bias of past experiences. For example, individuals who had mental health issues prior to COVID-19 had past experiences with virtual care visits, or had a delay in care in the past due to some reasons other than fear from COVID-19 are some examples that could have informed their present experiences, which could influence their responses. Additionally, the survey only captured delayed care and its impact on someone's health, and detailed survey information related to types of delayed care (eg, urgent or routine) was not included.

## Conclusion

This is a cross-sectional study, a provincial survey, to understand patient experiences during the pandemic and compare experiences among individuals born in Canada and outside of Canada. The study provides important insights from Albertan's healthcare experiences during COVID-19. The pandemic has increased the level of mental health issues, which was higher among Canadian-born individuals. Albertans reported an impact on their health due to delays in receiving care during the pandemic. A clear cost-effective community-based intervention, including better mental health care for everyone, is crucial to promote and enhance access to healthcare services. Virtual care is of growing interest, an expansion of investment in technology, training for the workforce in primary care settings, and educating patients about the virtual care process are unique opportunities to enhancing the quality of care and to improving care access.

The learning from this study would be a great opportunity for primary healthcare providers and policymakers to improve care quality and equitable access in PHC by better understanding diversity in populations. Vaccine hesitancy could be addressed by establishing strong communication strategies, implementing a mass vaccination campaign by involving key stakeholders, and mobilizing community leaders, which are crucial factors for consideration in successfully responding to the ongoing pandemic.

This study has informed the next research steps including an in-depth exploration of the experiences of individuals born outside of Canada and healthcare providers that will describe and identify gaps in accessing/delivering primary healthcare during COVID-19 from both patients’ and care providers’ perspectives.
